# Unraveling the Role of Spicules in Shaping Sponge Body Structure: Evidence from the Early Cambrian Shuijingtuo Formation

**DOI:** 10.3390/biology14070826

**Published:** 2025-07-07

**Authors:** Xinyi Ren, Yazhou Hu, Luke C. Strotz, Mei Luo, Caibin Zhang, Zhifei Zhang

**Affiliations:** 1 State Key Laboratory of Continental Dynamics, Shaanxi Key Laboratory of Early Life and Environments, Department of Geology, Northwest University, Xi’an 710069, China; 201810275@stumail.nwu.edu.cn (X.R.); eliyazhou@nwu.edu.cn (Y.H.); lukestrotz@nwu.edu.cn (L.C.S.); lm110072@163.com (M.L.); caibin-nwu@foxmail.com (C.Z.); 2 Department of Earth Sciences, Faculty of Geosciences, Utrecht University, 3584 CB Utrecht, The Netherlands

**Keywords:** sponge, Cambrian, micro-CT, micro-XRF, spicules

## Abstract

In most cases, sponge fossils are preserved as isolated spicules, with complete sponge body fossils largely confined to Konservat-Lagerstätten. Although the classification and diversity of sponges and their isolated spicules have been extensively studied, no systematic attempts have been made to define the relationship between fossil spicules and the sponge body plan. By utilizing relatively well-preserved sponge fossils from the black shales of the Shuijingtuo Formation (South China) in conjunction with isolated spicules from the same locality, we assess spicule morphology to identify the potential functional roles of spicules and chart their arrangement within the sponge body. The elemental distribution and three-dimensional morphology of the examined sponge body fossil (likely a hexactinelid) are assessed using both micro-XRF and micro-CT. Tetractine, stauractine and pentactine spicules are the most abundant spicule types, both in the body fossil and in acid residues, with an additional spicule type (monaxons) also present. The larger pentactine spicules (five-ray spicules) frame the structure, whereas the smaller tetractines and stauractines (four-ray spicules), along with smaller pentactines, are arranged along the branches of the larger spicules. Based on the arrangement of the different spicules, it is proposed that each of the spicule types represents a discrete functional form: monaxons support the overall sponge body plan, pentactines construct the framework of the parietal gaps, and the smaller pentactines or tetractines stabilize the framework of the parietal gaps. These results provide a new understanding of sponge morphology, spicule function and the relationship between isolated fossil spicules and associated sponge body fossils.

## Simple Summary

Complete fossils of sponges are typically only found in Konservat-Lagerstätten, with most fossil sponge remains instead consisting of discrete spicules. This study focuses on grid-like skeletal sponge fossils and associated discrete spicules from the black shales of the Shuijingtuo Formation (South China Yangtze Platform) to reconstruct the function and spatial arrangement of the different spicule types that make up the sponge skeleton. Morphological and functional analysis confirms the examined sponge material belongs to the Hexactinellids, with pentacines and stauractine spicules the dominant spicule types. The taxonomic affinity between macroscopic sponge specimens and isolated spicules is demonstrated in this paper. The spicule system exhibits clear functional zonation: monaxon spicules support the sponge body, pentactines construct the framework of the parietal gaps, and smaller spicules provide structural reinforcement. These results provide a new understanding of how spicule morphology contributes to the overall sponge body plan.

## 1. Introduction

Sponges (phylum Porifera) have played a crucial role in benthic marine communities from the Cambrian to the modern day [1]. Lacking both symmetry and true tissues [2], sponges are commonly considered an intermediate group between protozoans and other metazoans, serving as the sister taxon to the remaining Eumetazoa [3,4]. Sponge spicules have an easily identifiable needle-like form with multiple branches and three-dimensional structures. Because spicules are small in size and numerous in quantity, they are more likely to be preserved as fossils than the remains of other organisms, with acid maceration of suitable lithologies often yielding various forms of isolated spicules. As the most frequently identified fossils in the Cambrian fossil record, sponges constitute a critical data source for investigating early animal evolution, and the origin and evolution of early metazoans [5,6,7]. Isolated sponge spicules serve as an ideal tool for evaluating size variation, biomineralization processes, and the chemical composition of small shelly fossil assemblages [8,9,10]. The majority of fossil sponge spicules are extracted from limestones using acetic acid or identified in thin sections [4,10], but entire or partial sponge body fossils can be preserved under specific conditions, especially in black shales [11,12,13,14,15]. However, because often there is a lack of contrast between sponge body fossils and the surrounding matrix, important anatomical details are often not easily observed.

The morphology and taxonomy of sponge spicules have received considerable attention, as sponges are regarded as critical benthic sessile organisms during the early Cambrian period [5,6], and previous work includes a range of studies on the origin and evolution of fossil sponges that have provided valuable insights into the Cambrian Explosion [16,17,18,19,20,21,22,23,24,25,26,27,28]. However, the identification of sponge species has posed a significant challenge, as spicules often fail to provide sufficiently distinctive features for systematic classification [1]. Furthermore, following the acid-based extraction techniques typically used to obtain sponge material, the spicules become isolated elements, destroying any possibility of determining how the spicules were arranged. Thin-section analysis can go some way to helping with this issue, but the identification of sponge spicules in thin section is often contentious, and some researchers suggest that certain specimens might instead represent filamentous algae, mineral particles, or mineral crystals [16,29,30,31,32].

Using well-preserved sponge body fossils and isolated spicules from the Shuijingtuo Formation in South China, we herein analyze spicule arrangement for a sponge we identify as a Hexactinellid in an attempt to determine a more effective approach for assessing both sponge classification and spicule diversity. Based on our results, we reconstruct spicule positions within the sponge body plan and propose functions for each of the discrete spicule types, given evident differences in spicule distribution.

## 2. Geological Background

**Figure 1:**
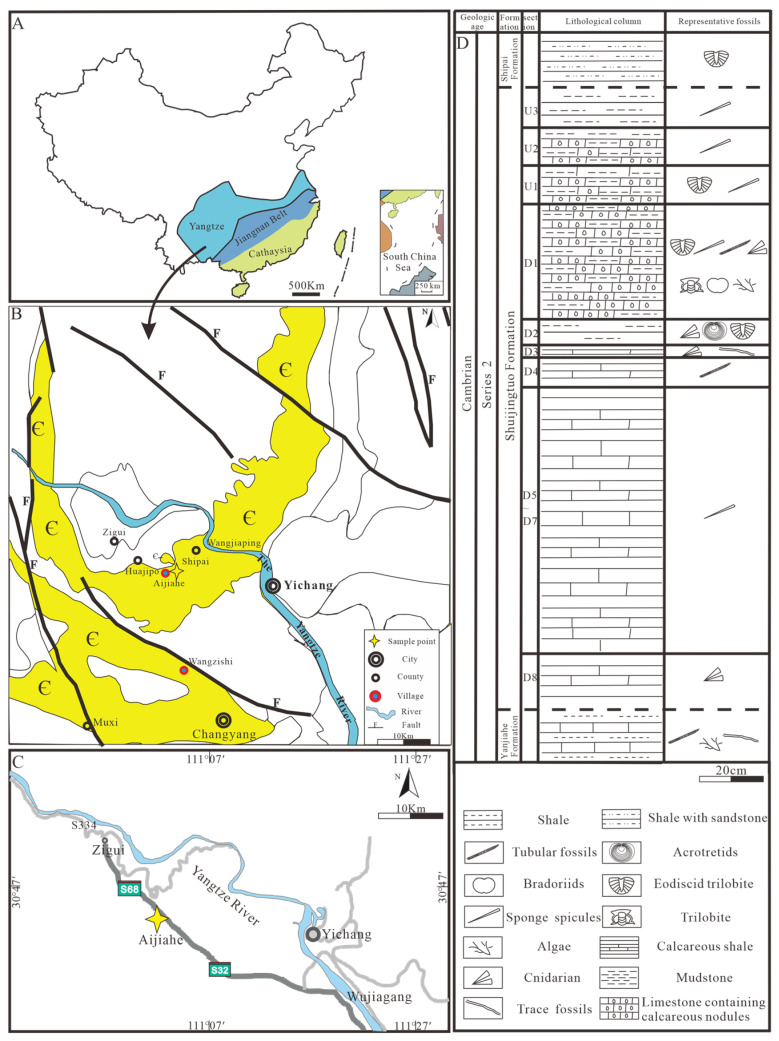
Regional geological map of China, map of sampling locations, partial geological map of Hubei Province, and stratigraphic column of the Shuijingtuo Formation in Aijiahe, Yichang. (**A**) Regional geological map of China, with the sampling area for this study located in Zone II. (**B**) Geological map of Yichang and its surrounding regions, with yellow stars highlighting the sampling points within the Cambrian strata (light yellow color). (**C**) Sampling site in Aijiahe, Yichang City, Hubei Province, indicated by the yellow star. (**D**) Stratigraphic column of the Shuijingtuo Formation for sample site near Aijiahe village.

The Yangtze Plate in China include relatively complete and accessible Cambrian strata, providing two crucial windows for investigating the evolution of early animal communities. Sedimentary strata from the Neoproterozoic–Cambrian, primarily found in the Three Gorges area on the southwestern flank of the Huangling Anticline, represent a continuous and extensively distributed sequence. The Neoproterozoic deposits consist of thick layers of Dengying Formation carbonate rocks, which are unconformably overlain by the lower Cambrian Yanjiahe Formation and Shuijingtuo Formation. The Yanjiahe Formation is approximately 40 m thick and primarily is made up of thin-to-medium beds of mudstone, banded mudstone, and limestone, interbedded with 1–2 cm thick layers of mudstone–sandstone mixtures [33]. The Shuijingtuo Formation, which unconformably overlays the underlying Yanjiahe Formation, consists of a sequence of deep-water carbonaceous shale layers (Figure 1) [34,35,36,37,38,39], mostly black calcareous shales interbedded with strip-shaped biogenic debris and argillaceous limestone, and is approximately 70 m thick. The Shuijingtuo Formation can be divided into three distinct parts. The bottom part (approximately 10 m thick) is characterized by multiple layers of limestone nodules, each with a diameter of about 1 m. These limestone concretions are well-developed and contain well-preserved small shelly fossils. The oldest trilobite, *Tsunyidiscus actus* Sun, appears approximately 3–5 m above the limestone nodules [34,35]. Above the nodules, there is a 40-m-thick layer of black shale interbedded with thin limestone, and the topmost layer consists of a 20-m-thick gray-to-black medium-to-thinly bedded argillaceous limestone. A diverse assemblage of shelly fossils has been reported from the Shuijingtuo Formation, including trilobites, brachiopods, archaeocyaths, and sponge spicules [34,35,40].

## 3. Materials and Methods

Abundant sponges and other skeletal fossil materials (tubular fossils, acrotretids, bradoriids, eodiscid trilobites, etc.) were collected from Aijiahe Village, Yichang City, western Hubei Province (Figure 1). This included approximately 1000 in situ preserved macro sponge fossils. The sponge fossils from the black calcareous shales were first examined under a binocular microscope (Zeiss, Oberkochen, Germany), and a Zeiss Smart Zoom 5 stereophotographic system (Zeiss, Oberkochen, Germany) was used to capture images. All fossils are preserved against a black background in argillaceous clastic rocks and calcareous limestones. Some sponge fossils occur as scattered spicule deposits, while others preserve complete morphological structures formed by spicules. Fossil preservation modes include pyritization, silicification, and calcite mineralization, among others. Some surfaces are covered with white calcareous films. Rock strata located near cultivated land are often weathered, resulting in the transformation of pyritized spicules into iron oxide. In contrast, rock strata far from cultivated land are more pristine, enabling the preservation of unoxidized three-dimensional pyrite spicules. The preservation of complete morphological structures indicates in situ deposition and burial, whereas the scattered accumulation of spicules may result from multiple processes, including post-depositional transportation.

The sponge spicules are preserved within limestones and limestone nodules and were treated with acetic acid at a concentration of 5–10%. The residues were extracted every three days, and the acetic acid solution was replaced with new acid with the same concentration as the previous treatment. The treatment temperature ranged from 18 °C to 32 °C. The residues were then washed several times and filtered through two sieves with mesh sizes of 0.07 mm and 2 mm. The residues were dried under sunlight or in an oven and examined under a microscope (ZEISS Stemi 305 microscope) to pick up individual spicules. Isolated spicules were coated with gold and imaged using SEM (scanning electron microscopy; Phenom XL G2, Phenom Scientific, Eindhoven, The Netherlands) [38]. One relatively intact sponge sample from the black calcareous shales was analyzed using micro-CT (micro-X-ray computed tomography; Phoenix v/tome/xm, ZEISS, Oberkochen, Germany) to non-destructively extract the three-dimensional sponge skeleton framework with spatial resolution of 35.89 μm. The micro-CT data were reconstructed using VG Studio (VG Studio max 3.2). Additionally, 91 samples from the black calcareous shales were examined using micro-XRF (micro-X-ray fluorescence spectrometry; M4 Tornado; Bruker, Karlsruhe, Germany) to identify elemental differences between sponge spicules and their surroundings. The area occupied by sponge spicules, as a proportion of the overall sponge body, was quantitatively assessed using micro-XRF images.

Sponge macrofossils were analyzed using micro-CT and micro-XRF to assess the morphology and map the elemental composition of the in situ spicules. SEM images of the isolated spicules extracted from acid residues were classified and organized into categories, based on their morphological characteristics, for further analysis. These SEM images were used for comparisons with the data obtained from micro-CT and micro-XRF analyses. For those spicule types that were successfully identified in both body fossils and acid residues, the micro-CT and micro-XRF datasets were further analyzed to determine the angles and positions of different spicules on the sponge body and to infer their potential functional roles.

The optical photograph, micro-CT and micro-XRF were also needed for defining the parietal gaps. The parietal gaps have different shapes, such as rhombuses, rectangles, sub-circles and so on. The centers of the parietal gaps are always blank on the surface of macrosponges, and they are decorated by a circle of spicules in different sizes and shapes.

Based on an extensive literature search, we compiled information on key sponge spicule character traits for modern sponges (55 Calcarea, 73 Demospongiae, and 324 Hexactinellida; see Appendix E, Appendix F and Appendix G). Traits included the number of spicule axes, radiation count, spicule morphology, and whether spicules develop from one center or multiple centers. Using these data, we performed a principal component analysis (PCA) to ascertain what higher-order clade our fossil sponge specimens belong to. To more intuitively demonstrate the morphological affinity between fossil spicules and modern clades, the Origin 8.5 software was utilized. Since PCA data is a reorganized dataset that reduces the dimensionality of the original data, it is sometimes difficult to accurately align the corresponding original data to the final result. The Origin software represents a suitable supplementary tool to address this issue. It features an image processing function that allows for direct superimposition of multiple datasets onto the same image, yielding more easily interpreted results. In essence, this involves projecting points from the original dataset onto a two-dimensional coordinate axis. By selecting key features such as the number of spicule rays and whether the developmental center is a single point, models of spicule types for the three modern sponge clades were constructed and compared with those observed in fossil samples. Specifically, we selected a single radiation point (coded as 1 for yes and 0 for no) as the Y-axis and the number of spicule rays as the X-axis. Each point on the associated plot represents the spicule parameters for a specific sponge type. All samples are currently stored at the Early Life Institute, Northwest University, China.

## 4. Results

### 4.1. Sponge Spicules Extracted from Limestones

**Figure 2:**
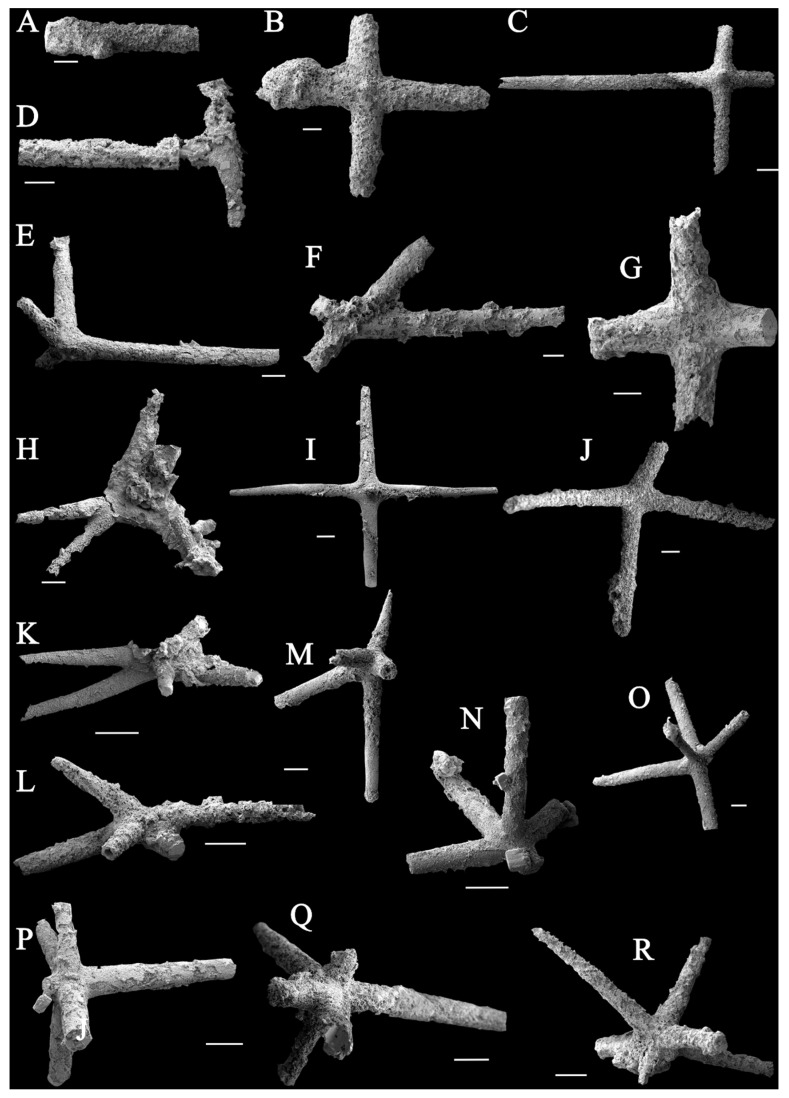
**Isolated sponge spicules** (**A**–**R**) (the scale bar represents 200 μm). Spicule (**A**) is rodlike and is incomplete (1 rays); Spicule (**D**) has 3 ends (3 rays); Spicules (**B**,**C**,**E**–**J**) have 4 branches in different directions (4 rays); Spicules (**K**–**O**) have 5 branches (5 rays); Spicules (**P**–**R**) have 6 branches (6 rays).

**Figure 3:**
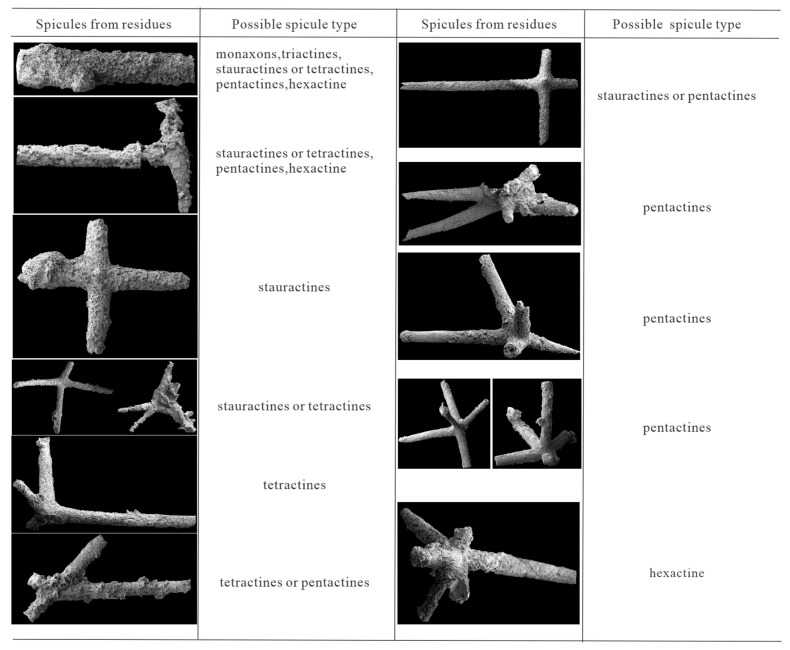
The possible spicule types recovered from acid residues.

The extracted spicules are preserved as pyrite, and they can be divided into those with four rays and those with five rays. Spicules with four rays can be classified into four groups: (1) Figure 2B,C and I represent standard cross-like spicules; (2) Figure 2G,J are spicules that develop on a curved surface; (3) Figure 2F,H form branches with obtuse and acute angles, respectively; (4) Figure 2E resembles a fork. Spicules with five rays can be divided into two groups: (1) Figure 2K,M are both X-shaped, with an additional branch diagonally intersecting the X-shaped plane; (2) Figure 2N,O are similar to Figure 2K,M, but developed on a curved surface, and the fifth branch is perpendicular to that surface (Figure 3).

**Figure 4:**
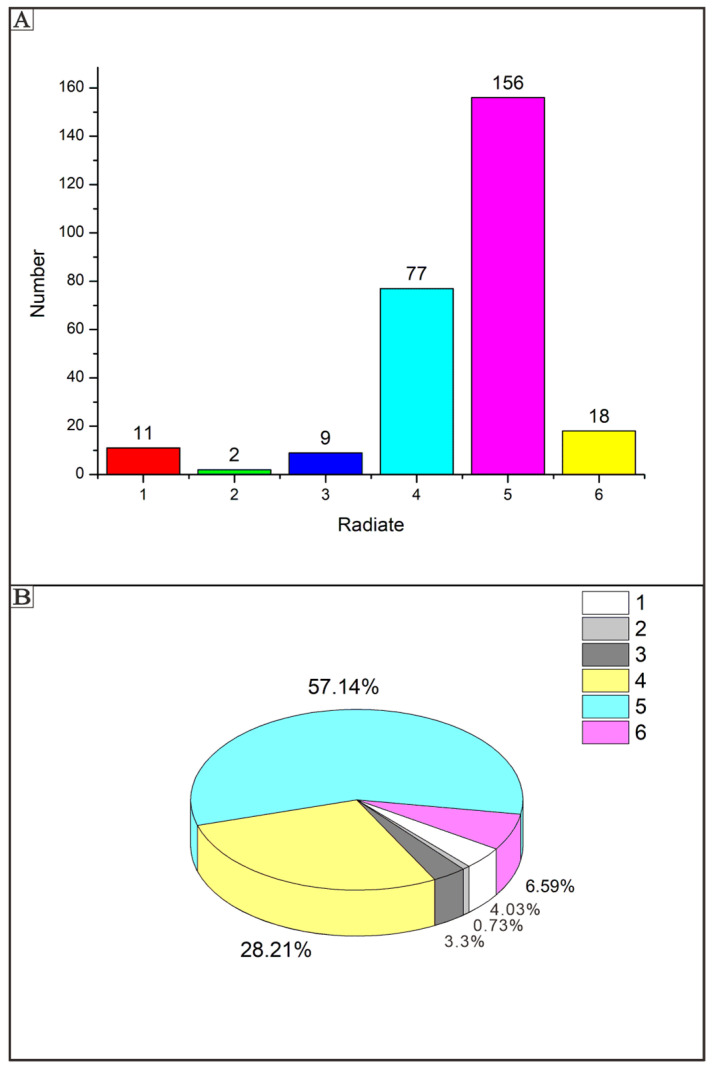
Proportions of each spicule form of isolated spicules presented as a column chart (**A**) and a pie chart (**B**). The 4-ray and 5-ray spicules represent the majority of spicules, with 5-ray spicules making up more than 50% of all specimens.

These six primary spicule types can also be grouped based on their axis and radiating structures. The possible range of forms of spicules from residues are displayed in Figure 3. The most abundant type is pentactine (Figure 2K,O; five rays in Figure 4). The second most common types are tetractines and stauractines (Figure 2B,J; four rays in Figure 4). Figure 2B,C,F,G,I,J seem to represent stauractines with rays arranged in a plane (consistent with Hexactinellids). Figure 2E,H appear to be true tetractines with a tetrahedral arrangement. The least abundant type is triactines (Figure 2D; three rays in Figure 4). The remaining types consist of monaxons (Figure 2A; single ray in Figure 4) and hexactines (Figure 2P,R; six rays in Figure 4).

### 4.2. Articulated Sponge Fossils Preserved in Black Shales

**Figure 5:**
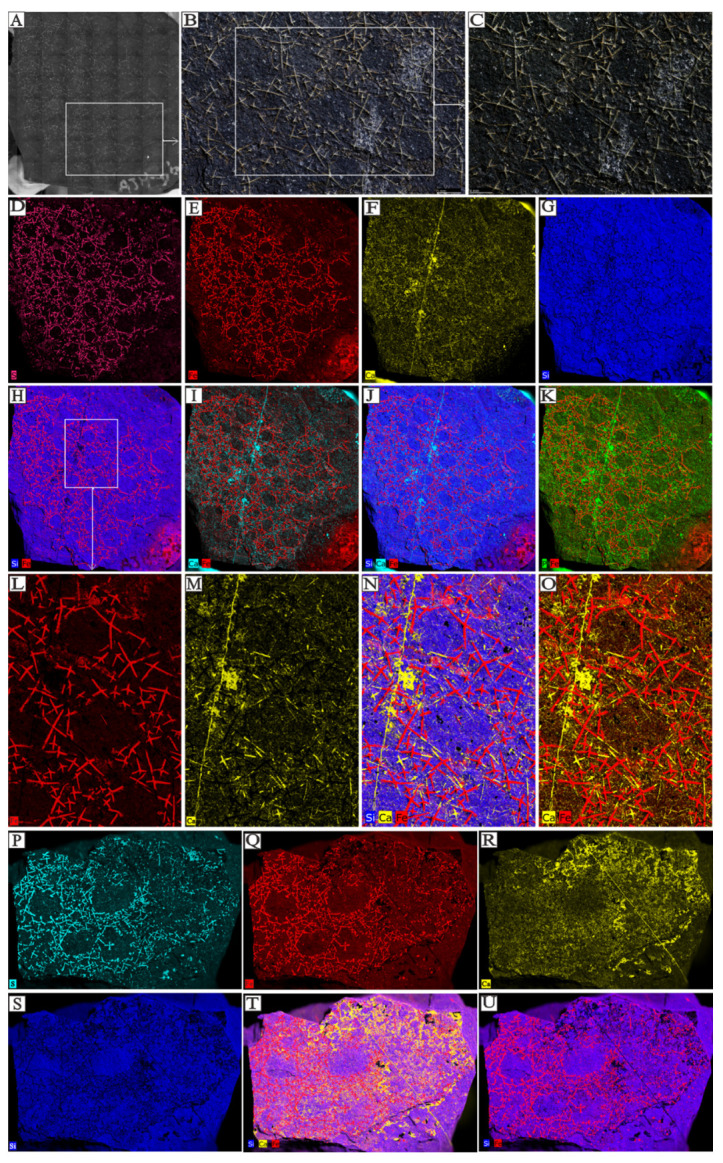
XRF elemental distribution of sponge spicules in the Shuijingtuo Formation, from Aijiahe village, Hubei province (sample number: Figures (**A**–**O**) are AJH-D6-213; red = iron, blue = silicon, russet = sulfur, green = phosphate, yellow = calcium; and Figures (**P**–**U**) are AJH-D5-184; red = iron, blue = silicon, sky blue = sulfur, green = phosphate, yellow = calcium). (**A**–**C**) show fossil morphology in situ. (**D**–**G**,**L**,**M**,**P**–**S**) show simple elemental information for the sponge fossils, and (**H**–**K**,**N**,**O**,**T**,**U**) show the distribution of multiple elements simultaneously for the same fossils. The sponge spicules consist of Ca, S, and Fe, with S and Fe often overlapping. The matrix consists of Si in all samples.

Based on micro-XRF analysis, iron and calcium elements were the primary elements selected for comparative analysis. The primary constituents of the spicules are sulfur, iron, and calcium, whereas the surrounding rock is predominantly composed of silica. Figure 5D–G depict the individual elemental distributions, while Figure 5H–K illustrate the range of elements associated with sponge spicule mineralization. Iron-based spicules are higher in abundance and more complex (in terms of branching patterns) compared to calcium-based spicules.

**Figure 6:**
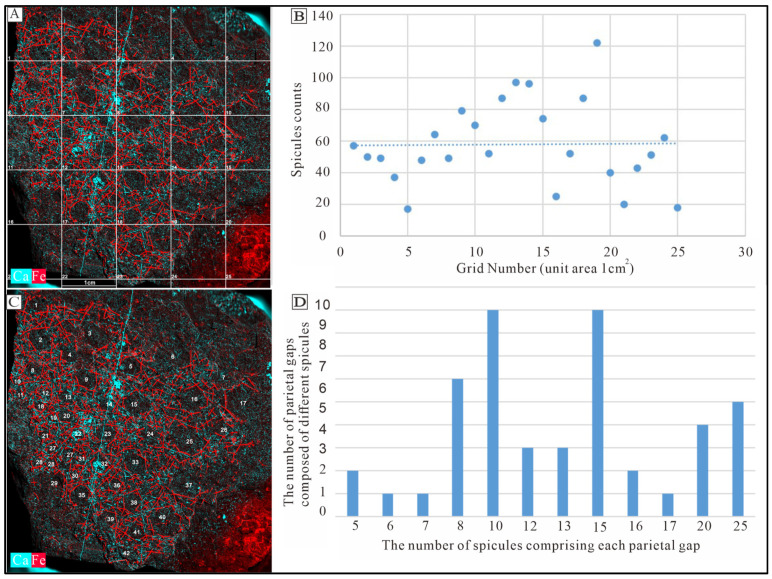
Iron (red color) and calcium (blue color) elements are compared based on micro-XRF elemental mapping. (**A**) illustrates the fossil divided into a 1 cm^2^ grid (1 cm width × 1 cm length). (**B**) depicts the number of spicules within each cell of the grid, where the horizontal axis represents each grid cell in order from left to right and top to bottom, and the vertical axis indicates the number of spicules in each corresponding grid cell. The majority of cells fall within the range of 40 to 80 spicules. (**C**) shows both the number of parietal gaps and their respective compositions, in terms of spicule count. (**D**) indicates that most parietal gaps are composed of 8, 10, 15, 20, or 25 spicules, while a few incurrent pores consist of only 6, 7, or 17 spicules.

**Figure 7:**
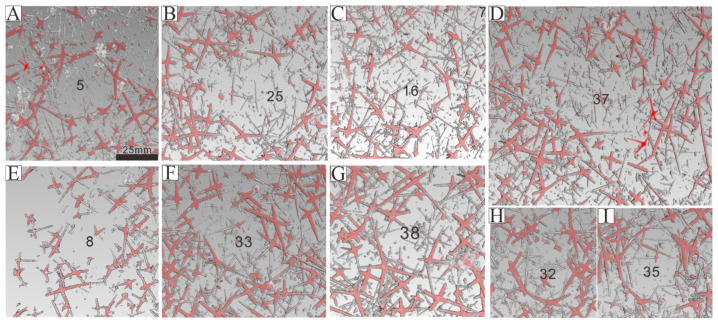
Micro-CT results. Parietal gaps (indicated by the numbers in black) are clearly surrounded by spicules (**A**–**I**). Micro-CT results indicating spicule counts for each parietal gap ranging from 5 to 29 (**A**–**I**, Appendix C), with the majority consisting of 10, 12, 15, or 16 spicules (Appendix C). All images are at the same scale.

**Figure 8:**
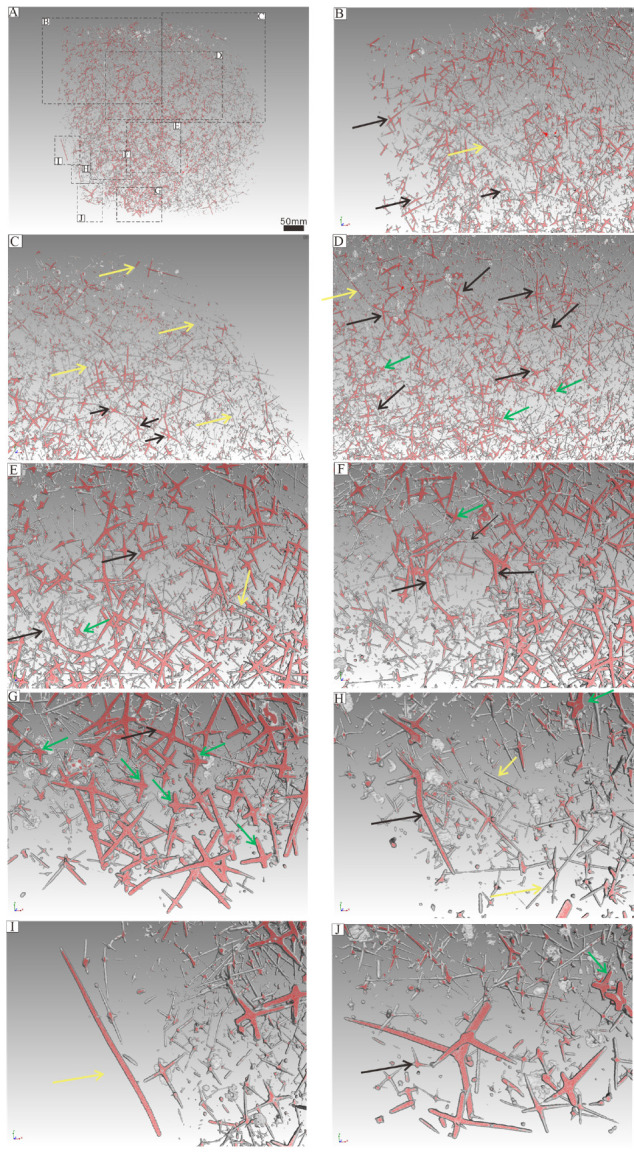
Micro-CT results showing spicule position and orientation for different spicule types associated with parietal gaps. Yellow, black, and green arrows are used to denote different types of spicules. Yellow arrows indicate monaxons, green arrows represent smaller five-ray spicules and black arrows signify larger three-/four-/five-ray spicules (Appendix A). (**B**–**J**) represent each of the labeled areas indicated in (**A**).

The in situ plate-like sponge macrofossil we examine in detail measures approximately 9 cm in length, 10 cm in width, and 1 cm in thickness. The sponge spicules are visible on the surface and are variable in both size and concentration. There are 40 to 80 spicules presenting in a 1 cm^2^ area (Figure 6A). Spicules are sometimes in a nested arrangement (in sets of two) or are arranged into structures consisting of four, six or seven spicules (Appendix C). As for the spicules recovered from acid residues, we are able to identify spicule types based on their morphology. These include monaxons, which are typically long and straight and often located on the outer side of the parietal gaps; larger pentactine (five-ray) and tetractine (four-ray) spicules, which construct the primary framework of the parietal gaps; and smaller pentactine, stauractine and tetractine spicules arranged along the branches of the larger spicules (Figure 7 and Figure 8, Video S1).

**Figure 9:**
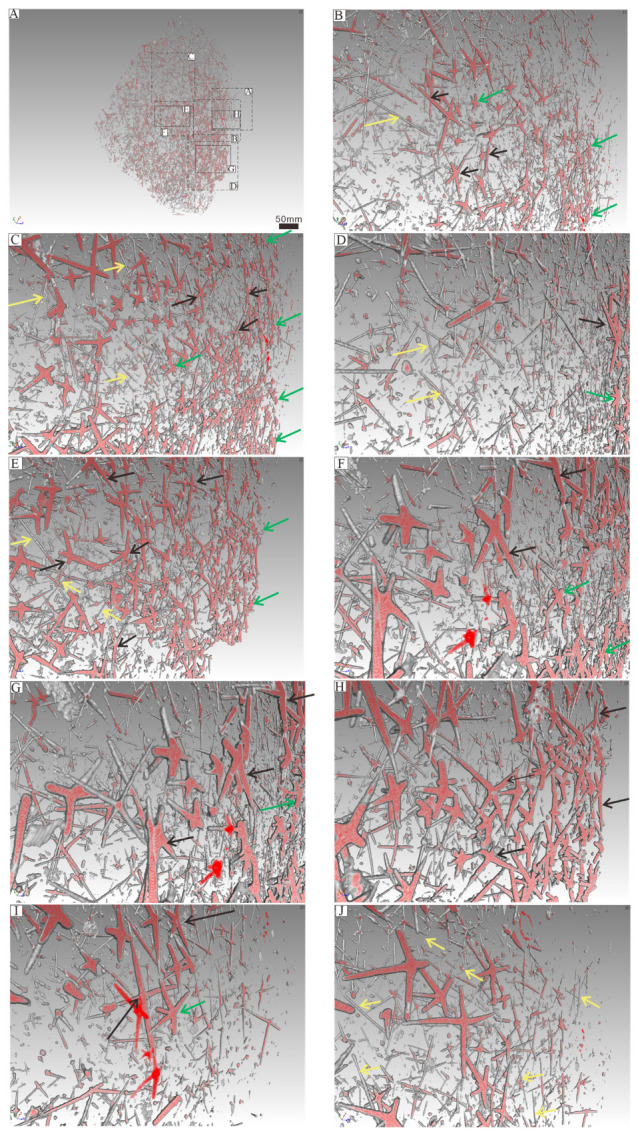
Micro-CT results showing spicule position and orientation for different spicule types associated with parietal gaps. (**A**–**J**) represent the same parts of the specimen depicted in Figure 8 but are rotated by 45 degrees to show the dominant orientations for each spicule in the third dimension. Yellow, black, and green arrows denote different types of spicules, with yellow arrows indicating monaxons, green arrows representing smaller five-ray spicules, and black arrows signifying larger three-/four-/five-ray spicules (Appendix A).

**Figure 10:**
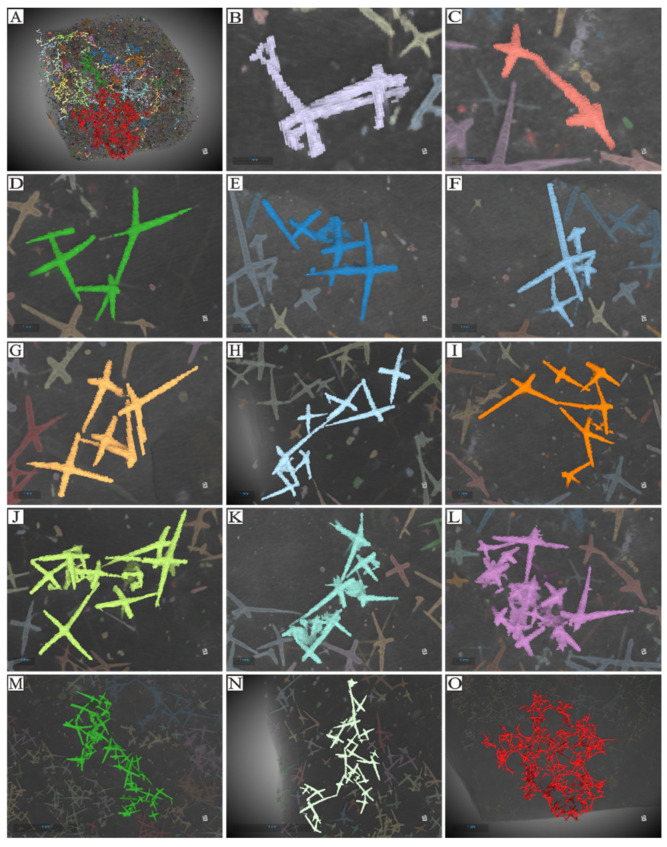
(**A**) Reconstructed Micro-CT data for the entire studied specimen. (**B**–**O**) Spicule arrangement for multiple spicule morphologies. Individual spicules are demarcated using false colors. The micro-CT reconstruction presented in (**A**) provides a preliminary delineation of the various types of spicules (see Appendix A), which are distinguished by different colors. The prominently colored regions are primarily composed of Fe. (**B**,**C**) depict a nested arrangement formed by two spicules. (**D**–**F**) illustrate a structure composed of four spicules. (**G**–**K**) show an arrangement of six or seven spicules. (**L**–**N**) show structures composed of multiple larger and smaller spicules. (**O**) illustrates multiple parietal gaps and the associated pattern of spicules. In (**D**,**F**–**K**), the meshwork of parietal gaps is composed of large spicules forming angles greater than 90 degrees. Although the angles are not uniform, the obtuse angles generally point toward each parietal gap, while the acute angles direct outward toward the tangential surfaces of the meshes. The construction modes can be categorized into three types: point–point contact, point–surface contact, and surface–surface contact. (**H**,**I**) show the branches of adjacent spicules converge at a certain point, representing point–point contact. In (**K**), one spicule’s branch inserts into the acute angle of an adjacent spicule, indicating point–surface contact. In (**B**,**J**), intersecting surfaces are formed by respective angles, signifying face–surface contact (Appendix A).

The parietal gaps are predominantly subcircular, differ in size, and are surrounded by varying numbers of spicules. Notably, the parietal gaps on the right side are larger than those on the left side, as depicted in Figure 5. Parietal gaps are surrounded by 5 to 29 spicules (Figure 5 and Figure 6, Appendix C), consisting of 1–8 larger pentactines that construct the parietal gap framework and 4–18 smaller pentactines, stauractines or tetractines that infill the area between these larger spicules. The spicules that make up the meshwork of parietal gaps form angles greater than 90 degrees (Figure 8 and Figure 9). Although the angles are not uniform, the obtuse angles generally point toward each parietal gap, while the acute angles point outward toward the tangential surfaces of the meshes. The spicules are connected in one of three ways (Figure 10): point–point contact (Figure 10C), point–surface contact (Figure 10D,G), and surface–surface contact (Figure 10B,E,F).

### 4.3. Spicule Morphological Affinities

**Figure 11:**
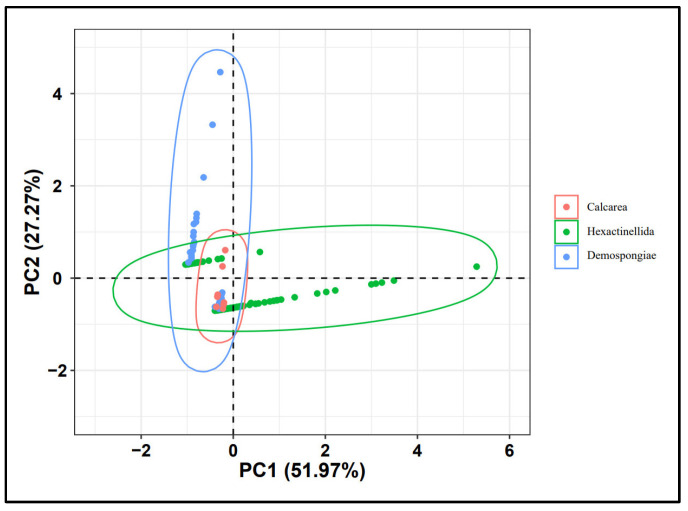
Principal Components Analysis for the three extant sponge clades: Calcarea (red), Hexactinellida (green) and Demospongiae (blue). Demospongiae and Hexactinellida are somewhat differentiated but Calcarea completely overlaps with Demospongiae and Hexactinellida.

**Figure 12:**
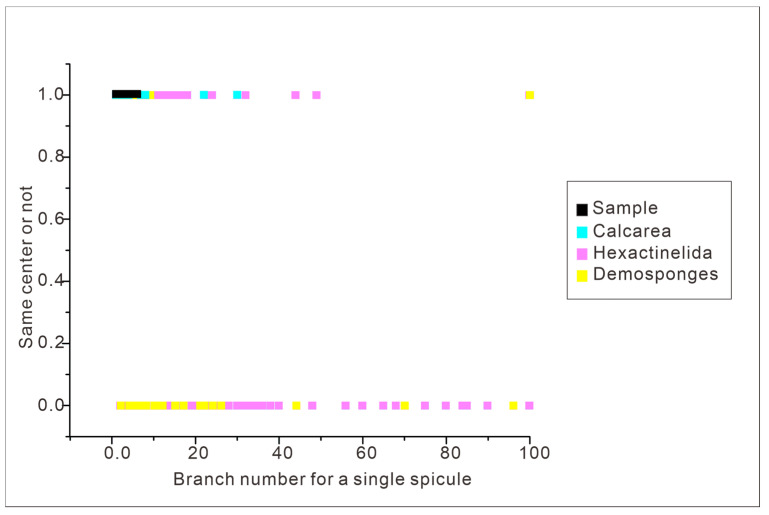
Results of Origin 8.6 analysis for extant sponge clades and our fossil sample. The horizontal axis represents the branch number for a single spicule from the relevant sponge clade. One the vertical axis, 0 represents spicules have more than one developmental center, and 1 means that spicules that have a single origin site.

The primary aim of this study is to correlate individual sponge spicules with their once-living source. However, we also aim to investigate whether it is possible to consistently determine sponge species based on isolated sponge spicules. Previous work has demonstrated that the morphology of sponge spicules serves as a valuable indicator for identifying sponge taxa [3,9,11,13,24]. Utilizing morphological characteristics for sponge spicules from 55 modern Calcarea, 73 modern Demospongiae, and 324 living Hexactinellida, obtained from previously published literature (see Appendix E, Appendix F and Appendix G), we identify the morphological variation for three modern sponge groups—Calcarea, Hexactinellida and Demospongiae—using principal component analysis (PCA) (Figure 11). Based on the results of our PCA, we determine that the three modern sponge clades (Calcarea, Hexactinellida, and Demospongiae) occupy distinct areas of morphospace, although with some overlap. We also conducted a similar analysis using the Origin 8.5 software. Our fossil specimens fall within the area represented by black points in the latter analysis (Figure 12). This area is occupied by all three sponge clades, making it impossible to precisely assign our specimens to a specific modern sponge type.

## 5. Discussion

### 5.1. Taxonomic Identification

It is not immediately apparent which clade the studied sponge material belongs to. Our analyses of spicule morphology only identifies that our specimen is unlikely to belong to the Calcarea. Calcarea typically possesses monaxons, triradiates, and tetractines that all radiate from the same location, which is not a feature we identify in our specimen (Appendix E, Figure 11 and Figure 12). Calcarea also produces a calcareous skeleton without microscleres [41,42]. We cannot be certain of the original composition of our fossil (as it has likely undergone some alteration), but our specimen does not have microscleres. Whilst Demospongiae (Appendix F, Figure 11 and Figure 12) can be traced back to the Cambrian period or earlier [43,44,45,46,47,48,49,50], they do not have spicules that are fused at the tips (which we do have in our specimen) (Figure 10J–L, Video S1). Demospongiae possess at least 12 types of megascleres, which are often coated and interconnected by spongin, and we do not have any evidence for this in our specimen. Hexactinellida (Appendix G, Figure 11 and Figure 12) are characterized by the presence of six-ray spicules, which are present in our specimen. Given this, it seems most likely that our sample should likely be classified as belonging to Hexactinellida [45,47]. After comparison of our specimens to Hexactinellida fossils from the Ordovician to Silurian periods and to the taxa in the modern Hexactinellida database maintained by the University of Tokyo (Appendix H) it is likely our material belongs to a Hintzespongid [50,51].

Results of PCA and Origin 8.5 analysis indicate that the use of disarticulated spicule fossils to directly determine the clade to which specimens belong is not a straightforward process. Consequently, isolated spicules from fossil assemblages are likely insufficient for defining sponge genera or species. Therefore, we suggest basing taxonomic determinations only on spicules should be undertaken with caution for Cambrian sponge taxa.

### 5.2. Spicule Orientation and the Function of Spicules in Sponge Framework Construction

Sponge spicule fossils can be preserved as different minerals under the influence of temperature, pressure and fluids [52,53,54,55]. Macroscopic sponge fossils and isolated spicules from the Shuijingtuo Formation are preserved in three-dimensions and consist of pyrite, indicating that the samples have not undergone excessive oxidation, which is similar to the composition of spicules in the Hetang Formation. This makes these specimens suitable for micro-CT scanning [51,56]. As is the case for many fossil sponges, the spongin [3], which links spicules, is not preserved, and the ostia are difficult to identify, given their size [51]. We thus focus herein exclusively on the skeletal structure of our specimen when assessing its construction and framework.

**Figure 13:**
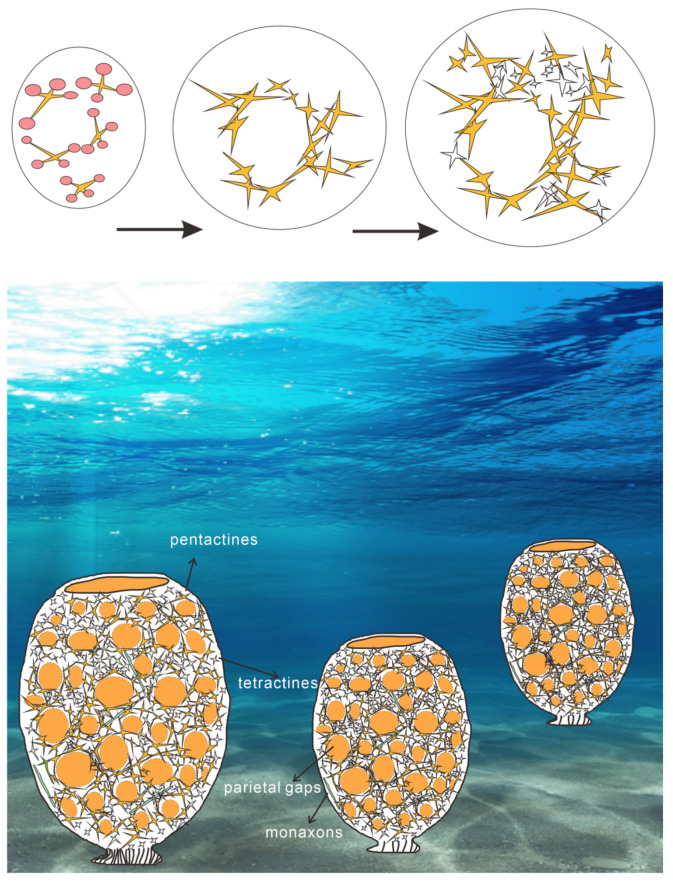
Reconstruction of Hexactinellida taxon from the Shuijingtuo Formation, eastern Three Gorges, Hubei Province (Reference from [13,24,41,45,48,51,57,58,59,60,61,62,63,64,65,66,67]. Copyright information for background photo is provided in Appendix I.

Given that monaxons are often located on the outer side of the parietal gap (Figure 8 and Figure 9), they likely serve as structural supports for the overall sponge body. The larger pentactines are oriented with their obtuse angle toward the blank gaps. The location and orientation of the larger pentactine and tetractine spicules indicate they are responsible for constructing the primary framework of the parietal gaps, and the smaller pentactine, stauractine and tetractine spicules then link this primary framework together, infilling the space between the larger spicules. Constructing the framework of the parietal gaps in our specimen requires 1–8 medium-sized spicules and 4–18 smaller spicules (Figure 6, Figure 7, Figure 8 and Figure 9, Appendix C). Whether this is typical of all sponges is impossible for us to currently say, given our parietal gaps are a unique shape compared to those identified in previous work. Prior investigations have identified parietal gaps that are diamond, triangle, or rectangle in shape, such as in *Cystospongia globosa* (Rigby, 1989) from the Chengjiang Fossil Lagerstätte [57],* Hintzespongia *(Rigby, 1983) and* Crumillospongia biporosa *(Rigby, 1986) from the Niutitang Biota [11,48,50,51,57,58,59,60] and Hintzespongiidae (Finks, 1983) from the Hetang Formation [48,51,58]. In our specimen, the parietal gaps are subcircular and are similar to those of the articulated sponge, Hintzespongiidae (Finks, 1983), from the Lower Cambrian Hetang Formation in southern Anhui, South China [51]. This Hintzespongid from the Hetang formation also has different sizes of pentactine spicules at the boundaries of ostia margins, with smaller spicules located at the outer margin of parietal gaps. Like in our specimen, the pentactines for this Hintzespongid are oriented with their obtuse angle toward the blank gaps [51]. This is additional evidence that our sponge fossil might belong to the Hintzespongid. We cannot say much about the concentration of spicules in the sponge body more generally (40–80 spicules per 1 cm^2^) as such values have not previously been compiled for fossil sponges. Based on the integrated analysis of micro-CT data, micro-XRF data, and assessment of isolated spicules, we provide a full reconstruction of the morphology of our sponge in Figure 13. 

### 5.3. Integrating Acid Residues with Macroscale Sponge Specimens

Results from acid residues indicate that pentactine, stauractine and tetractine spicules are the most abundant spicule types, which is consistent with the results obtained for the body fossil examined using micro-CT (Appendix C). This suggests that there is little to no loss of information regarding spicule proportions associated with either taphonomic processes or sample processing.

Based on their prevalence, it is evident that monaxons are not restricted to the parietal gaps. Instead, they can occur at any angle, in any part of the mesoglea outside the parietal gaps. We hypothesize that monaxons likely serve as a rigid support structure, supporting adjacent parietal gaps, and also prevent the collapse of mesoglea areas lacking reinforcement by other spicules. The larger pentactine and tetractine spicules primarily occur in the structural framework of parietal gaps. To maximize the size of the parietal gaps whilst minimizing the number of spicules needed, these spicules are not typically standard cross-shaped structures. Instead, they consist of X-shaped forms that are as flat as possible. This distinct morphology enables the construction of larger parietal gaps with fewer spicules interconnected. Given that spicule construction is likely an energy-intensive process, producing fewer spicules would thus likely enhance the overall fitness of individual sponges [68,69,70,71]. The smaller tetractines in the mesoglea are distributed around the framework of parietal gaps, analogous to the role of aggregates (such as gravel and sand) in concrete walls [72]. These aggregates increase the internal friction angle, enhancing the mechanical stability and overall strength of the structure. In the sponge mesoglea, if smaller tetractines were absent, the framework composed only of larger pentactines and tetractines would lack sufficient reinforcement. The smaller size of the tetractines allows them to occupy the interstitial regions of the mesoglea surrounding the larger spicules, stabilizing the adjacent parietal gaps. This structural arrangement likely plays a major role in enabling sponge choanocytes to perform regular filtration and feeding activities efficiently, as it maximizes the possible size of the parietal gaps through which such activities are conducted.

Although only a small amount of spongin was preserved in the analyzed sample, its role in the sponge skeleton should not be underestimated. Spongin, a type of keratin-like protein, functions similarly to adding countless fine fibers to concrete, enhancing both tensile and impact resistance. This reinforcement makes the sponge’s skeletal structure more robust and stable, contributing to the organism’s overall fitness [58].

### 5.4. The Significance of Combining Non-Destructive X-Ray Microscopy Techniques with Specimens from Acid Residues

One of the key innovations in this study is that, for the first time, spicules from acid residues were compared to fossil sponges preserved in situ from the same deposit. Additionally, the two- and three-dimensional morphology and chemical composition of the in situ fossils were extracted using non-destructive micro-XRF and micro-CT techniques. For the first time, three distinct spicule functional groups are identified in a single specimen. This was only possible because of the holistic approach we have utilized herein. We did find, however, that quantifying the number of spicules associated with each parietal gap can be influenced by whether one uses micro-XRF data or micro-CT data. Micro-CT provides a higher (and thus more accurate) spicule count, likely due to its higher resolution. Micro-XRF, despite its inability to fully depict the distribution of spicules, can still play a critical role in assessing sponge morphology. We found that a combination of the two techniques was the best way to discern the sponge from the surrounding matrix and was particularly important for accurately identifying parietal gaps (Figure 8 and Figure 9).

Carbonaceous rocks and clastic mudstone facies are among the most favorable lithologies for preserving a diverse array of fossils, including trilobites, corals, brachiopods, bivalves, molluscs, microdictyon, sponges and hyolithoids [52,53,54,55,56,73,74]. However, in such lithologies, these fossils can often only be obtained through acid extraction. Using this process disrupts the connectivity between the in situ organism and its fossil remains, limiting the possibility of high-resolution investigations. Even when in situ macrofossils can be identified, it may be difficult to properly distinguish their morphological features from the surrounding matrix, particularly when it comes to fine-scale structures. The methods we have used to study the sponge fossils from the Cambrian Shuijingtuo Formation serve as an exemplary case, addressing many of the above issues and offering a framework for studying in situ fossilized specimens, such as the numerous taxa that make up Cambrian Small Shelly Fossil faunas, in an articulated form.

## 6. Conclusions

This study represents the first comparison of sponge spicules from acid residues with in situ fossil sponges from the same deposit. The morphology and chemical composition of the in situ fossils were analyzed using non-destructive techniques (micro-CT; micro-XRF). Assessment of acid residues identified that spicules with 4 and 5 rays are the most abundant forms, a finding consistent with the assessment of spicule abundance for the associated macrofossils. Additionally, three distinct spicule functional groups are identified, based upon the position and abundance of the relevant spicules: monaxons act as overall support for the Hexactinellida sponge body; larger pentactines (1–8) construct the framework of the parietal gaps; and smaller pentactines, stauractines or tetractines (4–18) stabilize the aforementioned framework. This work provides a new approach to the assessment of sponge morphology that should facilitate comprehensive functional analyses of fossil sponges in the future.

## Figures and Tables

**Figure A1:**
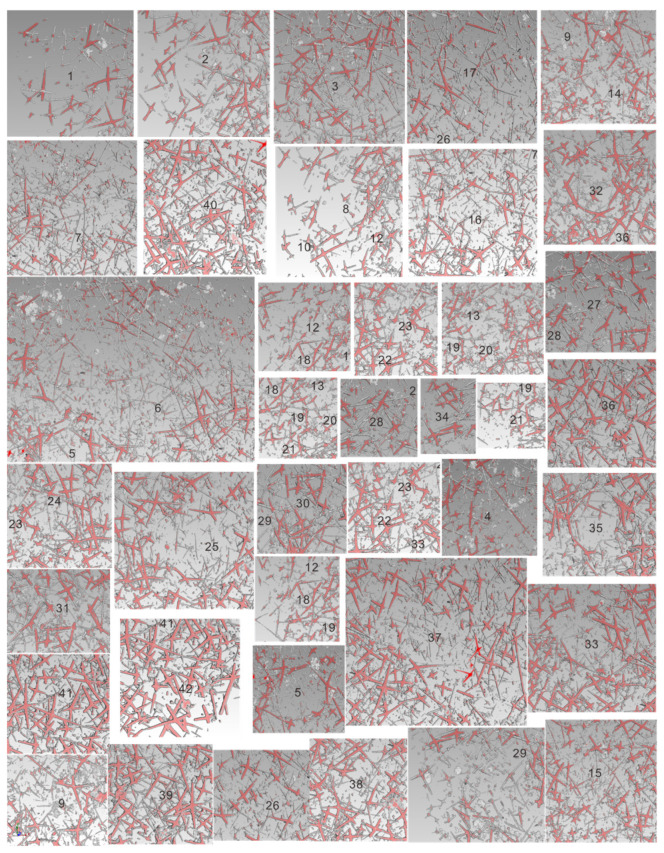
Morphology and spicule arrangement for the 42 parietal gaps (all images are at the same scale) based on micro-CT results.

## Data Availability

The original contributions presented in this study are included in the article. Further inquiries can be directed to the corresponding author.

## Supplementary Materials

The following supporting information can be downloaded at https://www.mdpi.com/article/10.3390/biology14070826/s1, Video S1: reconstruction of micro-CT data from sponge spicules from the Shuijingtuo Formation.

## Abbreviations

The following abbreviations are used in this manuscript:

SEM	scanning electron microscope
XRF	X-ray fluorescence spectrometer
PCA	principal component analysis

## Appendix A

Video S1 reconstruction of micro-CT data from sponge spicules from the Shuijingtuo Formation.

## Appendix B

**Table A1:** Names of sponge spicules based on morphological classification. (x × y: x represents axes number, y represents ray number) (modified from [5,23,24,41,42,43,44,45,46,47,48,62,63,64,65,66,68,69].

Number	X × Y	Name
1	1 × 1	monaxons diactine
2	2 × 2	bixon triactines
3	2 × 3	bixon triactines
4	2 × 4	bixon tetractines
5	3 × 3	triaxon triactines
6	3 × 4	triaxon tetractines
7	3 × 5	triaxon pentactines
8	3 × 6	triaxon hexactines/hexactinestrixons
9	4 × 4	tetraxon tetractines
10	4 × 5	tetraxon pentactines
11	5 × 5	pentaxon pentactines
12	6 × 6	hexactines
13	three forks at the top with one long rod	triaenes
14	Uniform 2 × 4	orthotetractines
15	T-shaped, like 2 × y	T-form bixones
16	Multi-rayed	polyactinals

## Appendix C

**Table A2:** The spicule number for the 42 parietal gaps.

No.	Sponge Spicule Number (Micro-XRF)	Bigger Spicules (Parietal Gaps Framework) (Micro-XRF)	Smaller Spicules (Linkage of Parietal Gaps Framework) (Micro-XRF)	Sponge Spicule Number (Micro-CT)
17	5	1	4	5
6	13	2	11	13
11	6	2	5	7
1	12	3	9	12
7	7	3	4	7
9	13	3	10	13
12	17	3	14	17
13	10	3	16	19
22	15	3	12	15
26	8	3	6	9
30	10	3	9	12
31	8	3	8	11
34	15	3	12	15
38	10	3	9	12
2	15	4	18	22
4	10	4	6	10
15	25	4	24	28
18	10	4	6	10
19	10	4	6	10
21	10	4	6	10
24	16	4	12	16
27	15	4	11	15
28	12	4	8	12
33	20	4	16	20
36	25	4	9	13
37	20	4	16	20
41	20	4	16	20
42	10	4	18	22
8	16	5	11	16
16	15	5	16	21
10	8	5	3	8
20	8	5	9	14
23	25	5	24	29
35	15	5	10	15
3	25	6	18	24
25	15	6	12	18
29	15	6	18	24
32	12	6	10	16
39	10	6	10	16
14	15	7	10	17
40	25	7	18	25
5	20	8	13	21

## Appendix E

**Table A3:** Spicule types in modern Calcarea.

No.	Spicules’ Axis Number	Spicules’ Radiate Number	Spicules Developed from the Same Center (Yes = 1, No = 0)	Spicules Developed from Different Centers (Yes = 1, No = 0)
16	0	0	0	0
1	1	1	1	0
4	1	1	1	0
5	1	1	1	0
7	1	1	1	0
9	1	1	1	0
11	1	1	1	0
12	1	1	1	0
19	1	1	1	0
20	1	1	1	0
21	1	1	1	0
22	1	1	1	0
23	1	1	1	0
24	1	1	1	0
6	2	2	1	0
1	3	3	1	0
2	3	3	1	0
3	3	3	1	0
4	3	3	1	0
5	3	3	1	0
6	3	3	1	0
7	3	3	1	0
8	3	3	1	0
9	3	3	1	0
10	3	3	1	0
11	3	3	1	0
12	3	3	1	0
13	3	3	1	0
14	3	3	1	0
15	3	3	1	0
17	3	3	1	0
19	3	3	1	0
20	3	3	1	0
21	3	3	1	0
22	3	3	1	0
23	3	3	1	0
24	3	3	1	0
1	4	4	1	0
2	4	4	1	0
4	4	4	1	0
6	4	4	1	0
12	4	4	1	0
15	4	4	1	0
19	4	4	1	0
20	4	4	1	0
21	4	4	1	0
22	4	4	1	0
23	4	4	1	0
24	4	4	1	0
4	7	7	1	0
4	8	8	1	0
4	22	22	1	0
4	30	30	1	0

## Appendix F

**Table A4:** Spicule types in modern Demospongiae.

No.	Spicules’ Axis Number	Spicules’ Radiate Number	Spicules Developed from the Same Center (Yes = 1, No = 0)	Spicules Developed from Different Centers (Yes = 1, No = 0)
1	1	1	1	0
2	1	1	1	0
3	1	1	1	0
4	1	1	1	0
6	1	1	1	0
7	1	1	1	0
8	1	1	1	0
9	1	1	1	0
10	1	1	1	0
11	1	1	1	0
12	1	1	1	0
13	1	1	1	0
14	1	1	1	0
2	2	2	1	0
6	2	2	0	1
7	2	2	0	1
8	2	2	0	1
12	3	3	1	0
13	3	3	1	0
1	4	4	1	0
4	4	4	1	0
8	4	4	0	1
10	4	4	0	1
10	4	4	1	0
11	4	4	0	1
12	4	4	1	0
14	4	4	0	1
3	5	5	0	1
8	5	5	1	0
12	5	5	1	0
13	5	5	0	1
13	5	5	1	0
16	5	5	1	0
1	6	6	1	0
2	6	6	0	1
10	6	6	1	0
10	6	6	1	0
11	6	6	0	1
12	6	6	0	1
16	6	6	1	0
8	7	7	1	0
8	7	7	0	0
10	7	7	1	0
12	7	7	0	1
13	7	7	0	1
15	7	7	0	1
1	8	8	1	0
8	8	8	0	1
10	8	8	1	0
12	8	8	0	1
14	8	8	1	0
14	8	8	0	1
12	9	9	1	0
15	9	9	1	0
13	10	10	0	1
12	11	11	0	1
13	11	11	0	1
13	11	11	0	1
14	12	12	0	1
9	15	15	0	1
14	15	15	0	1
15	15	15	0	1
8	17	17	0	1
5	21	21	0	1
10	22	22	0	1
9	24	24	0	1
6	26	26	0	1
8	26	26	0	1
10	44	44	0	1
10	70	70	0	1
7	96	96	0	1
10	200	200	1	0

## Appendix G

**Table A5:** Spicule types in modern Hexactinellida.

No.	Spicules’ Axis Number	Spicules’ Radiate Number	Spicules Developed from the Same Center (Yes = 1, No = 0)	Spicules Developed from Different Centers (Yes = 1, No = 0)
1	1	1	1	0
2	1	1	1	0
3	1	1	1	0
5	1	1	1	0
6	1	1	1	0
7	1	1	1	0
9	1	1	1	0
11	1	1	1	0
13	1	1	1	0
23	1	1	1	0
6	2	2	1	0
9	2	2	0	1
2	2	3	1	0
3	3	3	1	0
7	3	3	1	0
10	3	3	1	0
11	3	3	1	0
12	2	3	1	0
14	2	3	1	0
20	3	3	1	0
22	2	3	1	0
23	3	3	1	0
25	3	3	1	0
30	3	3	1	0
1	4	4	1	0
2	2	4	1	0
3	4	4	1	0
5	2	4	1	0
5	3	4	1	0
6	4	4	1	0
6	2	4	1	0
6	2	4	1	0
7	4	4	1	0
7	4	4	0	1
7	4	4	1	0
8	4	4	1	0
9	4	4	0	1
10	2	4	1	0
14	2	4	1	0
15	2	4	1	0
19	2	4	1	0
21	2	4	1	0
22	2	4	1	0
23	4	4	1	0
23	2	4	1	0
26	3	4	1	0
30	2	4	1	0
30	4	4	1	0
31	2	4	1	0
32	3	4	1	0
36	2	4	1	0
1	3	5	1	0
4	5	5	1	0
5	4	5	1	0
6	3	5	1	0
7	3	5	1	0
7	5	5	1	0
7	5	5	1	0
7	5	5	1	0
7	5	5	1	0
10	5	5	1	0
11	3	5	1	0
11	5	5	1	0
13	3	5	1	0
15	3	5	1	0
17	3	5	1	0
20	3	5	1	0
21	3	5	1	0
22	3	5	1	0
23	3	5	1	0
23	5	5	1	0
24	3	5	1	0
25	3	5	1	0
27	3	5	1	0
28	3	5	1	0
29	3	5	1	0
30	3	5	1	0
30	4	5	1	0
33	3	5	1	0
35	3	5	1	0
1	3	6	1	0
2	3	6	1	0
3	5	6	1	0
3	6	6	1	0
4	6	6	1	0
4	3	6	1	0
5	6	6	1	0
5	3	6	1	0
5	6	6	1	0
6	3	6	1	0
6	46	6	0	1
6	6	6	1	0
7	3	6	1	0
7	6	6	1	0
7	6	6	1	0
7	6	6	1	0
7	6	6	0	1
8	3	6	1	0
10	6	6	1	0
11	3	6	1	0
11	6	6	0	1
13	3	6	1	0
13	6	6	0	1
15	3	6	1	0
16	3	6	1	0
17	3	6	1	0
18	3	6	1	0
19	3	6	1	0
20	3	6	1	0
21	3	6	1	0
23	3	6	1	0
27	3	6	1	0
28	3	6	0	1
28	6	6	0	1
29	3	6	1	0
29	3	6	1	0
33	3	6	1	0
34	3	6	1	0
35	3	6	1	0
36	3	6	1	0
5	7	7	0	1
7	7	7	1	0
7	7	7	1	0
7	7	7	0	1
7	7	7	1	0
7	7	7	0	1
8	7	7	0	1
8	7	7	1	0
10	6	7	0	1
11	7	7	1	0
11	7	7	1	0
12	7	7	1	0
23	4	7	0	1
28	7	7	0	1
5	8	8	0	1
5	8	8	0	1
6	7	8	0	1
7	8	8	0	1
7	8	8	1	0
7	8	8	1	0
7	8	8	0	1
8	8	8	0	1
11	8	8	1	0
17	8	8	1	0
17	8	8	1	0
20	8	8	1	0
23	8	8	1	0
30	8	8	0	1
32	8	8	0	1
3	9	9	1	0
3	9	9	0	1
4	9	9	1	0
4	9	9	0	1
5	9	9	0	1
6	8	9	0	1
6	9	9	0	1
7	9	9	1	0
7	9	9	1	0
7	9	9	1	0
7	9	9	1	0
8	9	9	0	1
8	9	9	1	0
11	9	9	1	0
12	9	9	1	0
26	9	9	0	1
33	9	9	0	1
3	10	10	1	0
4	10	10	1	0
5	10	10	0	1
5	10	10	0	1
6	7	10	0	1
7	10	10	0	1
7	10	10	1	0
8	10	10	0	1
11	10	10	0	1
32	10	10	0	1
5	11	11	0	1
6	11	11	0	1
6	10	11	0	1
7	11	11	1	0
7	11	11	1	0
7	11	11	0	1
11	11	11	1	0
12	11	11	1	0
13	11	11	0	1
24	11	11	0	1
2	12	12	0	1
4	12	12	0	1
5	12	12	0	1
6	12	12	0	1
7	12	12	1	0
7	12	12	0	1
7	12	12	1	0
8	12	12	0	1
10	12	12	0	1
11	12	12	0	1
14	12	12	0	1
15	12	12	0	1
20	12	12	0	1
21	12	12	1	0
22	12	12	0	1
25	12	12	0	1
4	13	13	0	1
5	13	13	0	1
7	13	13	1	0
7	13	13	1	0
7	13	13	1	0
23	13	13	0	1
29	13	13	0	1
32	13	13	0	1
4	14	14	0	1
6	14	14	0	1
7	14	14	1	0
7	14	14	1	0
10	13	14	0	1
17	14	14	0	1
20	14	14	0	1
26	14	14	0	1
35	14	14	0	1
36	14	14	0	1
3	15	15	0	1
4	15	15	0	1
7	15	15	0	1
8	15	15	1	0
10	13	15	0	1
25	15	15	0	1
5	16	16	0	1
6	16	16	0	1
7	16	16	1	0
8	16	16	0	1
14	16	16	0	1
15	16	16	0	1
19	16	16	0	1
20	16	16	0	1
25	16	16	0	1
30	16	16	0	1
4	17	17	1	0
4	17	17	0	1
6	17	17	0	1
4	18	18	0	1
6	18	18	0	1
7	18	18	1	0
8	18	18	0	1
11	18	18	0	1
4	19	19	0	1
19	19	19	0	1
30	19	19	0	1
35	19	19	0	1
6	20	20	0	1
7	20	20	0	1
10	20	20	0	1
11	20	20	0	1
12	20	20	0	1
16	20	20	0	1
22	20	20	0	1
5	21	21	0	1
11	21	21	0	1
24	21	21	0	1
7	22	22	0	1
14	22	22	0	1
19	22	22	0	1
32	22	22	0	1
7	23	23	1	0
11	23	23	0	1
23	23	23	0	1
1	24	24	1	0
2	24	24	0	1
4	24	24	0	1
20	24	24	0	1
25	24	24	0	1
26	24	24	0	1
29	24	24	0	1
6	25	25	0	1
36	25	25	0	1
11	26	26	0	1
24	26	26	0	1
10	27	27	0	1
7	28	28	0	1
7	28	28	0	1
6	30	30	0	1
27	30	30	0	1
30	30	30	0	1
11	31	31	0	1
12	32	32	0	1
16	32	32	1	0
19	33	33	0	1
4	34	34	0	1
23	35	35	0	1
2	36	36	0	1
17	36	36	0	1
26	36	36	0	1
19	38	38	0	1
36	38	38	0	1
27	40	40	0	1
19	44	44	1	0
10	48	48	0	1
12	48	48	0	1
16	48	48	0	1
12	49	49	1	0
13	56	56	0	1
19	56	56	0	1
6	60	60	0	1
7	60	60	0	1
30	35	65	0	1
24	68	68	0	1
19	75	75	0	1
13	80	80	0	1
29	80	80	0	1
17	84	84	0	1
23	85	85	0	1
13	90	90	0	1
19	100	100	1	0
19	108	108	0	1
13	138	138	0	1
13	150	150	0	1
12	162	162	0	1
19	210	210	0	1
12	216	216	0	1
19	224	224	0	1
19	240	240	0	1
12	350	350	0	1
19	432	432	0	1
17	640	640	0	1

## Appendix H

The modern Hexactinellida database is maintained by the University of Tokyo (https://umdb.um.u-tokyo.ac.jp/DDoubutu/invertebrate_en/porifera/specimens/index.html) (accessed on 7 January 2025).

## Appendix I

The ocean photo is from https://pngtree.com/freebackground/blue-seabed-creative-texture-background_1173063.html, (accessed on 13 November 2024). The author has obtained the commercial license from the copyright owner.
